# Sensory temporal sampling in time: an integrated model of the TSF and neural noise hypothesis as an etiological pathway for dyslexia

**DOI:** 10.3389/fnhum.2023.1294941

**Published:** 2024-01-03

**Authors:** Oliver H. M. Lasnick, Fumiko Hoeft

**Affiliations:** brainLENS Laboratory, Department of Psychological Sciences, University of Connecticut, Storrs, CT, United States

**Keywords:** developmental dyslexia, reading disorder, neural noise hypothesis, temporal sampling, TSF, neural oscillations, entrainment

## Abstract

Much progress has been made in research on the causal mechanisms of developmental dyslexia. In recent years, the “temporal sampling” account of dyslexia has evolved considerably, with contributions from neurogenetics and novel imaging methods resulting in a much more complex etiological view of the disorder. The original temporal sampling framework implicates disrupted neural entrainment to speech as a causal factor for atypical phonological representations. Yet, empirical findings have not provided clear evidence of a low-level etiology for this endophenotype. In contrast, the neural noise hypothesis presents a theoretical view of the manifestation of dyslexia from the level of genes to behavior. However, its relative novelty (published in 2017) means that empirical research focused on specific predictions is sparse. The current paper reviews dyslexia research using a dual framework from the temporal sampling and neural noise hypotheses and discusses the complementary nature of these two views of dyslexia. We present an argument for an integrated model of sensory temporal sampling as an etiological pathway for dyslexia. Finally, we conclude with a brief discussion of outstanding questions.

## 1 Introduction

Developmental dyslexia or decoding-based reading disorder (DD or decoding-based RD) is estimated to have a prevalence of 5–10% (Peterson and Pennington, 2015). It is marked by a specific (not explainable by factors such as low IQ or poor vision) impaired ability to match word forms to their accompanying speech sounds (referred to broadly as “decoding skills”). Dyslexia is frequently comorbid with language disorders, attention-deficit hyperactivity disorder (ADHD), auditory processing disorders, other specific learning disorders [e.g., writing disability (dysgraphia) and mathematics (dyscalculia)], as well as internalizing disorders like anxiety and depression (King et al., 2003; Sharma et al., 2006, 2009; Nelson and Harwood, 2011; Nelson and Gregg, 2012; Peterson and Pennington, 2015; Barker et al., 2017; Hendren et al., 2018; Langer et al., 2019; Giovagnoli et al., 2020). The long-standing view was that the primary contributing factor to dyslexia are phonological deficits: deficits in skills related to the identification and parsing of speech sounds, especially in relation to the pronunciation of novel words or pseudowords (Fox and Routh, 1980; Vellutino and Scanlon, 1987; Manis et al., 1993; for a meta-analysis on phonological skills in reading see Melby-Lervåg et al., 2012). This gave rise to the phonological “core deficit” model of dyslexia (Snowling et al., 1997).

While phonological awareness (PA) has remained one of the most common characteristics of dyslexia in the modern research landscape, the dominant view is increasing adherence to the *multiple deficit model* (MDM). The MDM states that behavioral manifestations of complex disorders are premeditated by multiple cognitive deficits, each of which is affected by multiple etiological risk factors (Pennington, 2006; Van Bergen et al., 2014). There are substantive neuroimaging findings which report widely distributed neural markers for distinguishing typical readers from those with dyslexia (Richlan, 2020). In addition, results from family history, twin, and molecular genetics studies have elucidated clear yet diverse genetic contributions to the disorder (Schumacher et al., 2007; Kere, 2014; Gialluisi et al., 2021). Researchers are increasingly subscribing to the view that dyslexia is cognitively and genetically multifaceted, with multiple avenues for the conferral of risk: it is heterogeneous and based on an individual's unique genetic and environmental profile (Pennington, 2006; Van Bergen et al., 2014). However, while the etiology of dyslexia is not currently fully understood, there have been several neurobiological theories proposed.

One view of dyslexia implicates *neural oscillations*, which reflect the combined electrical activity of populations of neurons (Buzsáki and Draguhn, 2004). Two viewpoints which touch on the role of oscillations in dyslexia are the temporal sampling framework (TSF) (Goswami, 2011) and the neural noise hypothesis (NNH) (Hancock et al., 2017). Briefly, the former describes impairments in language (primarily auditory speech) processing as a causal precursor to dyslexia and identifies their possible electrophysiological correlates (abnormal neural entrainment to speech); the latter more fully characterizes the etiology of the disorder, ranging from key genetic and neurochemical sources for atypical development (etiological factors) to observed electrophysiological endophenotypes. In both cases, these theories make note of the observed differences in neural processing of language stimuli between those with and without dyslexia.

This review will provide an overview of both the TSF and the NNH. We will first cover the theoretical and empirical evidence on the nature of dyslexia from the perspective of these two theories. For the TSF, this will consist primarily of an updated literature review. We consider the review of empirical research in the NNH to be especially important since there have not been any comprehensive reviews or evaluations of original research (related to the NNH specifically) published at time of writing. Second, we will integrate the two perspectives into a coherent etiological framework. This paper's position is that these two frameworks are complementary due to their mutual focus on auditory speech sampling in time as it relates to dyslexia. The TSF views the role of early language processing deficits in the development of dyslexia as being of paramount importance, while the NNH starts from the genetic and neurochemical properties of the brain. This review will further highlight the overlap between the two frameworks.

## 2 Two prominent neurobiological theories of dyslexia

### 2.1 Preface: neural oscillations and entrainment

Neuronal populations that are located spatially close to one another in the brain often display collective (group-level) dynamical behavior. This is demonstrated by the correlation between an individual neuron's spiking activity and the aggregate population-level activation (Tkačik et al., 2014; Okun et al., 2015; Gu et al., 2019). *Neural oscillations* are a formal description of this phenomenon, in which populations of neurons generate rhythmic, repetitive activity, creating a summed electrical potential which is detectable on the scalp (Buzsáki and Draguhn, 2004; Fries, 2005). This group-level activity is recorded when we use imaging methods such as electroencephalography or magnetoencephalography (EEG/MEG).

*Neural entrainment* occurs when populations of neurons become tuned to external stimuli. When this happens, the rhythmic activity of neuronal populations may become optimized to process this input. Such stimulus-locked activity is often referred to as “evoked” activity; non-stimulus locked activity that is still caused by exogenous rhythms is referred to as “induced” (Obleser and Kayser, 2019). We refer to intrinsic activity in the absence of stimuli as “endogenous” activity. The ability for neurons to entrain to stimuli could be crucial for cognitive tasks requiring the use of working memory, visual, and auditory processing (Tallon-Baudry et al., 1998; Jensen and Tesche, 2002; Kaiser et al., 2003; Joris et al., 2004). As an example, an external stimulus may be a rhythmic auditory signal such as speech. Entrainment is posited to optimize the neurons' speech encoding by increasing excitability at crucial time points in the signal (Lakatos et al., 2007, 2008). Neurons' ability to encode significant acoustic properties of speech is therefore enhanced (Arnal and Giraud, 2012).

### 2.2 The temporal sampling framework for dyslexia

One biological theory on the development of dyslexia is the TSF, which originally implicated abnormalities in processing at low-frequency (delta and theta) bands as early neural markers for later reading and language deficits. Within this framework atypical oscillatory activity exerts downstream causal effects on phonemic parsing (Goswami, 2011). It has been suggested that in children who go on to develop dyslexia, there is abnormal entrainment to prosodic (slow-wave delta range, ~1−4 Hz), and syllabic (slow-wave theta range, ~4–10 Hz) frequencies in speech (Goswami et al., 2010; Gross et al., 2013; Leong and Goswami, 2014). This is posited to result in a “cascade of impairment” characterized by disrupted processing at phonemic frequencies, which then manifests behaviorally as impaired phonological processing. Within this framework, deficient prosodic-rate speech perception is reflective of an impaired ability to recognize relative dominance (stress patterns) of the component syllables; and deficient syllabic-rate temporal integration of speech affects phoneme perception by disrupting the integration of relevant acoustic features. The main culprit within this framework, then, is disrupted evoked and possibly even induced activity, resulting in a reduced ability to phase-lock to speech stimuli.

Since 2011 there have been several topical reviews (Gnanateja et al., 2022; Stein, 2023) published which have both provided additional support to the TSF as well as proposed elaborations (Calderone et al., 2014; Goswami, 2019), modifications (Vidyasagar, 2013; Goswami et al., 2014; Pammer, 2014; Archer et al., 2020), and/or criticisms (Protopapas, 2014). Higher-level gamma oscillations (ranging typically from ~20 to 50 Hz) specialized for the tracking of phonemes have been suggested to play a role in the development of dyslexia (Giraud and Poeppel, 2012; Giraud and Ramus, 2013; Vidyasagar, 2013; Kershner, 2020). Visual correlates, related to the entrainment of visual cortical networks at spatial frequencies, have also been proposed (Goswami et al., 2014; Pammer, 2014; Archer et al., 2020). Some researchers have also argued that genetics studies should be able to provide evidence of an early gene-to-brain pathway, as multiple “dyslexia genes” have been identified (Kere, 2014; Gialluisi et al., 2021) and may contribute to early auditory processing and entrainment [see comment by Seidenberg (2011); and Giraud and Ramus (2013)]. We discuss this literature in more detail below (“Critiques of past theories & our proposal”).

In summary, an external stimulus may induce a change in the magnitude or timing of a neuronal signal, either decreasing or increasing excitability at a given moment. Neuronal populations in the language network may entrain to language stimuli starting very early in life, and disruptions could cause a cascade of language processing deficits that culminate in disorders of both language and literacy acquisition.

### 2.3 The neural noise hypothesis of dyslexia

The NNH proposes that excessive cortical “noise” (variability in neural activity at the individual and network levels) within the reading/language network of the developing brain disrupts network synchrony. It has been argued separately that a certain level of stochastic firing within networks is critical to preserve normal cognitive processing (Solanka et al., 2015); the NNH clarifies that excessive neural noise could be maladaptive, disrupting network synchrony. This synchrony is necessary for efficient processing of multisensory representations. It is the driving force for oscillations, as neurons must synchronize their activity in order for them to encode sensory stimuli (often in multiple modalities) and then bind them together into a coherent percept.

The NNH also suggests a contribution of altered neurochemistry—an imbalance of the excitatory (glutamate/Glu) and inhibitory (gamma-aminobutyric acid/GABA) neurotransmitter systems—to abnormal oscillatory behavior in neuronal populations, and argues that dyslexia “risk genes” may contribute to low-level sensory processing deficits and deficits in print-speech integration via effects on cortical excitability (Hancock et al., 2017). Imbalanced excitatory and inhibitory neurotransmitter concentrations and the resulting neural noise/cortical synchrony (indexed by oscillatory activity) are testable endophenotypes for dyslexia. The NNH thus offers a plausible direct link between genes and behavior that is mediated by cortical network synchrony.

In summary, the NNH consolidates research on distal contributors to endophenotypes associated with dyslexia. These endophenotypes—hyperexcitability caused by elevated glutamatergic activity and/or abnormal neural migration—are endogenous properties of the “dyslexia brain”, and may serve as the foundations for disrupted entrainment (evoked/induced activity) to speech as per the TSF.

### 2.4 Critiques of past theories & our proposal

Both the TSF and the NNH implicate atypical cortical oscillations as a potential biomarker for dyslexia. The TSF suggests that impaired auditory sampling early in life leads to impaired phonological representations and reading impairment. The NNH implicates cortical hyperexcitability (an endophenotype with plausible links to neurotransmitter and genetic profiles) as an underlying causal factor for altered neuronal dynamics, which interferes with multisensory integration and eventually phonological awareness. The connection between these two frameworks is straightforward: the former emphasizes early-life contributions from auditory language processing; the latter posits an etiological origin for dyslexia at the levels of genes, neurochemistry, and cortical excitability, all of which have been implicated in dyslexia. In both frameworks excessive cortical noise can be viewed as a contributor to atypical endogenous neural dynamics, resulting in disrupted evoked activity. However, there have been many additional questions and criticisms regarding the theoretical framework on the contribution of neural oscillations to dyslexia which have emerged in the years since the TSF was first published (Table 1).

**Table 1:** Brief description of reviews, editorials, and comments published since 2011 which discuss neural noise and oscillations in dyslexia.

**References**	**Modality**	**Summary/Conclusions**
**Goswami (** **2011** **)**	Auditory	Original proposal for the TSF. Characterizes dyslexia by disruption in temporal sampling of slower acoustic modulations in speech.
Seidenberg (2011)	Auditory	Argues for more comprehensive study in the TSF framework, starting from the level of genes and early development.
Giraud and Poeppel (2012)	Auditory	Summarizes empirical results from studies of speech-tracking at delta, theta, and gamma rates on overall language processing.
Giraud and Ramus (2013)	Auditory	Connects dyslexia neurogenetics to auditory delta/theta (1–7 Hz) and low-gamma (20–40 Hz) oscillations via abnormal cortical microcircuitry and connectivity.
Vidyasagar (2013)	Visual	Suggests top-down visuospatial attentional processes affect oscillatory activity during reading. Argues for deficit of impaired sampling at low-gamma rate.
Protopapas (2014)	Auditory	Critical review of the TSF. Asserts that more precise and empirical accounts of proposed causal mechanisms and explanations of heterogeneity are required.
Pammer (2014)	Visual	Proposes visual correlate of the TSF; oscillations in visual cortex at key spatial frequencies affect perception in ways that create dyslexia symptoms.
Calderone et al. (2014)	Audiovisual	Review arguing that neural entrainment to temporal structure of attended stimuli underlies selective attention, with implications for dyslexia and ADHD.
Goswami et al. (2014)	Visual	Editorial discussing visual correlates to the TSF and offering scientific standards for establishing effects of sensory processing on reading outcomes.
**Hancock et al. (** **2017** **)**	Audiovisual	Original proposal of the NNH. Suggests a distal cause of dyslexia: overexcitability resulting in imprecise timing between multisensory cortical networks.
Goswami (2019)	Auditory	Reaffirms neural entrainment to speech at low frequencies affects speech intelligibility and development of phonological awareness. Establishes updated oscillatory-linguistic hierarchy for speech processing (2–20 Hz).
Kershner (2020)	Auditory	Review of neural oscillations and lateralization in dyslexia; left-hemisphere networks specialize in processing high-frequency gamma rhythms in speech, while right-hemisphere networks process lower-frequency rhythms.
Archer et al. (2020)	Visual	Further extends TSF to visual domain; implicates abnormal theta oscillations in visual cortex during reading as a visual correlate for dyslexia.
Gnanateja et al. (2022)	Auditory	Short review of neural oscillations in speech and music processing; neural oscillations in dyslexia reflect atypical cortical tracking of speech input.
Stein (2023)	Audiovisual	Reviews past/current theories of dyslexia. Concludes symptoms are attributable to imprecise timing of multimodal signals (auditory/visual) during reading.

Bolded author names indicate the original texts for the TSF/NNH papers.

Early comments on Goswami's proposed TSF suggested that it lacked a clear connection to theory regarding dyslexia etiology, such as genetic factors and effects during early neural development; as well as justifications for elevated rates of comorbidity and heterogeneity in the disorder (Seidenberg, 2011; Protopapas, 2014). Some researchers pursued this line of thinking in subsequent years, with Giraud and Ramus (2013) drawing a theoretical link between the neurogenetics of dyslexia and low-frequency neural oscillations in the auditory modality. It is plausible that common dyslexia “candidate genes” and other genes implicated in language affect the development of cortical circuitry and rhythmicity (Giraud and Ramus, 2013; Murphy and Benítez-Burraco, 2018). Low-gamma oscillations were also later proposed by multiple researchers to be abnormal in dyslexia, due to their relevance to processing speech stimuli at the phonemic rate (Giraud and Ramus, 2013; Kershner, 2020); and to visuospatial processing and attention, which may be affected in dyslexia (Vidyasagar, 2013; Calderone et al., 2014; Pammer, 2014). This is in spite of the original TSF proposal emphasizing the selective contribution of low-frequency oscillations to dyslexia, and comparatively unimpaired gamma sampling (Goswami, 2011). Yet to the best of our knowledge, there has not been a large-scale attempt to integrate these broad and occasionally disparate perspectives into a single framework, and most empirical work on temporal sampling and neural oscillations focus primarily on characterizing endophenotypes.

Another important criticism of the neural oscillation literature as it relates to language processing (and reading) is present in a response to Giraud and Poeppel (2012) by Obleser et al. (2012). While the criticism is not aimed explicitly at the TSF, the authors' conclusions are still relevant to this area of research. They assert that specific frequency bands are not always well-defined across the literature, and further that the distinct timescales present in speech itself may not perfectly mirror the intrinsic ones in the brain. This casts a reasonable amount of doubt on the generalizability of neural oscillation research at the level of individual studies, and as such broad theoretical conclusions based on study data should be drawn with an appropriate degree of caution.

One of the main issues we believe can be addressed by integrating the TSF and the NNH is the etiology of dyslexia. We agree with Seidenberg (2011) that the TSF, even in its more updated version(s), is not comprehensive regarding its explanation of the origin of dyslexia. Some of the core principles of the NNH, given its increased emphasis on genetic contributors to neural endophenotypes, can do much to fill this gap. Furthermore, we hope to bring together some of the disparate perspectives and elaborations on temporal sampling as it relates to dyslexia—in particular, the contributions of visual correlates and various functional roles ascribed to different frequency bands—into a coherent, streamlined theoretical model. Within this integrated framework, endophenotypes of excessive cortical noise predispose children toward disrupted entrainment to multiple hierarchical linguistic structures in speech (which are encoded by different frequency bands); these hierarchical levels include phonemes, syllables, and prosodic structure (Luo and Poeppel, 2007; Ghitza and Greenberg, 2009; Soltész et al., 2013; Rimmele et al., 2021). There are therefore multiple crucial junctures in language processing, at all levels of this linguistic hierarchy, that could be associated with dyslexia. Variation in this process may emerge both within and between individuals (developmental effects vs. individual differences).

We therefore propose an integrated model of the TSF and the NNH, with the goal of (1) presenting a more explicit testable hypothesis on the etiology of developmental dyslexia, as well as (2) addressing some of its more common behavioral correlates and comorbidities.

## 3 An integrative model of sensory temporal sampling as an etiological pathway for dyslexia

**Figure 1:**
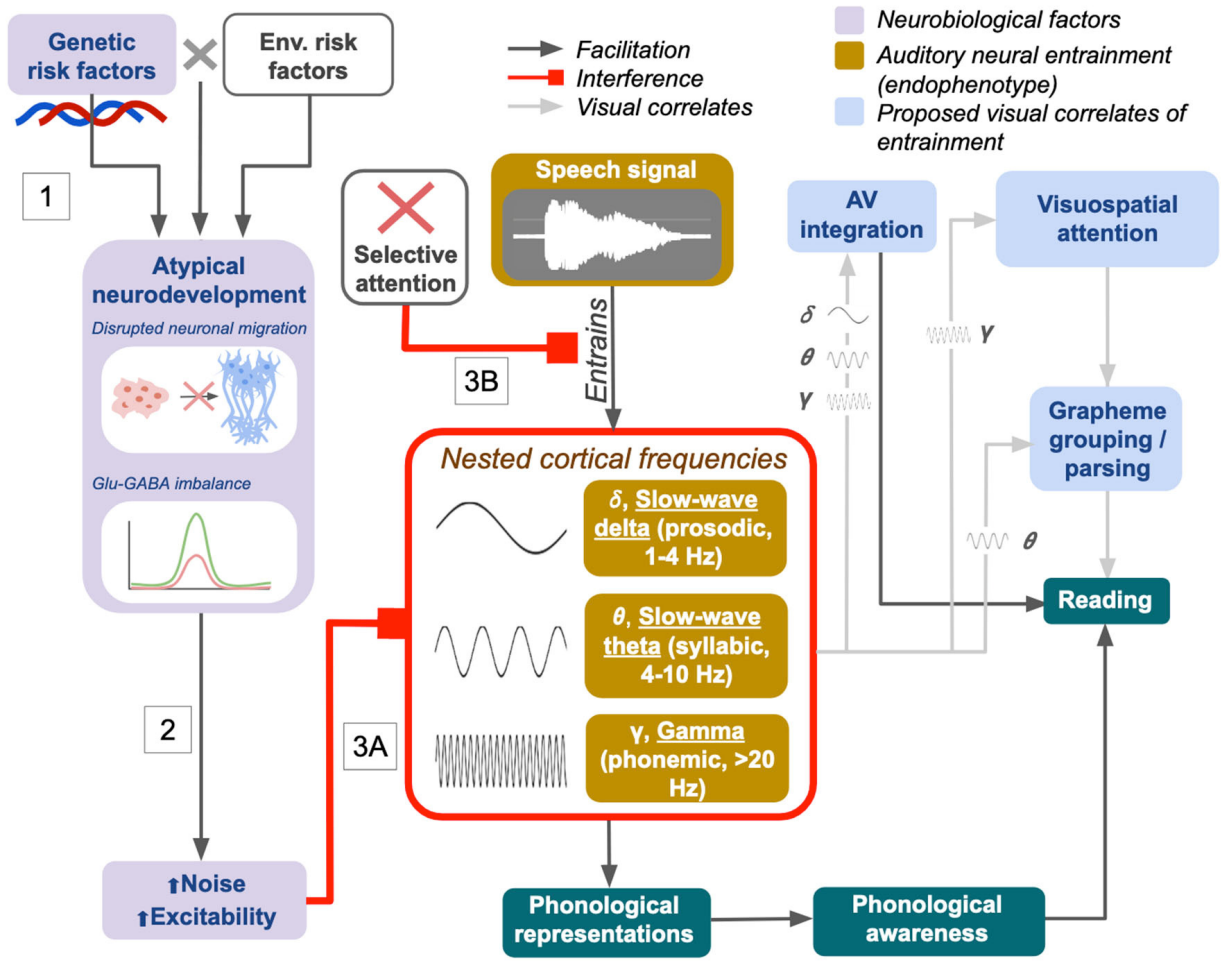
Visual summary of the proposed neurobiological mechanisms/contributors for dyslexia in the integrated NNH/TSF. Figure is adapted from the flowcharts presented in both original texts: Goswami (2011, p. 5) and Hancock et al. (2017, p. 437).

Current research suggests it is plausible that the integration of hierarchical speech information at multiple temporal timescales and the contribution of modulatory visual cues are both crucial for reading acquisition. One must be able to effectively parse rapid auditory stimuli to fluently process speech. High sensitivity to background noise, deficits in noise suppression, and endogenous neural noise which interferes with speech encoding could all be causal risk factors for language and reading disorders. With this in mind, we here propose a holistic view of dyslexia based around the concept of sensory temporal sampling (Figure 1).

### 3.1 Genetic and environmental risk factors

Figure 1 illustrates how relative contributions from genetic **(G)** and environmental **(E)** risk factors and G × E interactions during early life may result in impoverished language exposure, altered brain development (i.e., migration, neurochemistry imbalance/hyperexcitability), or both (Figure 1, Box 1). Studies on potential risk genes for dyslexia have shown associations with increased expression of Glu receptor genes, spontaneous receptor activity and neurotransmitter release, cortical spike timing variability, and morphology of various gray and white matter structures; some are also thought to be involved in early cellular developmental processes such as neuronal migration and dendritic/axonal formation (Kere, 2014; Marino et al., 2014; Eicher et al., 2016; Hancock et al., 2017; Murphy and Benítez-Burraco, 2018; Gialluisi et al., 2021).

Overall genetic risk is likely conferred via one of many possible complex polygenic profiles, which may include a subset of the identified candidate genes for dyslexia or perhaps none (intergenerational multiple deficit model, or iMDM; Van Bergen et al., 2014). Just as important to consider are environmental risk factors, such as early life stress, lower socioeconomic status, or an impoverished home literacy environment: disadvantageous environments can increase one's susceptibility to risk genes or constitute a possible developmental pathway to dyslexia in their own right (Theodoridou et al., 2021).

### 3.2 Neurodevelopmental trajectories

In Figure 1, the path from atypical neurodevelopment to increased neural noise and excitability (Figure 1, Box 2) represents the proposed pathway described by the NNH. Elevated Glu and E-I imbalances cause hyperexcitability and interfere with white matter tract growth via effects on precise spike timing and synchronized neuronal activity. Differences in white matter microstructure may arise because of long-term effects on synaptic plasticity between neurons: if action potentials fire inconsistently due to increased levels of neural noise and spike timing variability, this could have long-term effects on the establishment of synaptic connections. Altered patterns of connectivity may then co-occur with altered white matter micro- and macro-structures in the reading/language network, or even in low-level or subcortical sensory regions involved in early acquisition of language skills (Rimrodt et al., 2010; Goswami, 2011; Pajevic et al., 2014; Krishnan et al., 2016; Hancock et al., 2017; Beaulieu et al., 2020).

Figure 1 ultimately demonstrates how the cumulative effects of genes and environment on cortical excitability affect entrainment to language input in the auditory domain at varying timescales (Figure 1, Box 3A). An analogous effect on temporal sampling in the visual domain may result in a subset of dyslexic children having visual processing deficits, while others may show only impaired AV integration (Goswami, 2011; Van Bergen et al., 2014; Hancock et al., 2017).

The roles of attention and memory in learning to read are worth discussing here as well. Dyslexia and ADHD are frequently comorbid (Hendren et al., 2018), and the level of sustained selective attention is correlated both with reading fluency and phonological awareness (Guerra et al., 2023). It has been proposed that selective attention to aspects of one's environment, including speech, relies on effective entrainment (Calderone et al., 2014). Disrupted entrainment may serve as a neural mechanism for attentional deficits (Obleser and Kayser, 2019), suggesting a common underlying factor that could help explain elevated comorbidity between dyslexia and ADHD; at the same time, because selective attention is a crucial ability for facilitating speech processing (Calderone et al., 2014), attentional deficits can compound pre-existing issues with entrainment or disrupt entrainment in their own right, further contributing to reading deficits. This possibility is presented in Figure 1, Box 3B. Similarly, deficits in phonological working memory and working memory more generally have been noted in dyslexia (Schuchardt et al., 2008; Szenkovits et al., 2016; Cowan et al., 2017); and verbal working memory (digit span) deficits in participants with dyslexia have been found to be associated with enhanced higher-frequency entrainment beyond 40 Hz in left hemisphere language regions (Lehongre et al., 2011). Working memory load is also known to correlate with oscillatory activity in the theta, alpha, and gamma ranges, making its role in dyslexia consistent with the deficits observed from the perspective of sensory temporal sampling (Roux and Uhlhaas, 2014; Wilsch and Obleser, 2016).

### 3.3 Individual differences

How can an integrated account of temporal sampling and neural noise account for individual variability in outcomes? First, we must acknowledge that there are many factors—both genetic and environmental—that confer risk or protection to different individuals (see previous 2 sections). At the level of genetics, certain genes may be more or less important to the development of reading skills; and they can vary in how susceptible their expression is to the effects of an impoverished environment. Environmental factors vary considerably in terms of family dynamics and available financial or educational resources. A key question is whether individual differences in etiological factors (G, E, G × E) map to individual differences in neural endophenotypes associated with excess noise and disrupted entrainment, and in turn whether these differences can account for phenotypic variation in reading ability and dyslexia. We propose that this is indeed the case: that there is significant covariation between phenotypic measures (such as phonological awareness/PA, phonological decoding, and reading composites) and underlying measures such as (1) neurotransmitter concentrations (Bruno et al., 2013; Pugh et al., 2014) and (2) continuous indices of neural entrainment (Power et al., 2013; De Vos et al., 2017a,b, among others).

In the next section we will expand on empirical evidence for the central claims of this overarching theory, starting from genetic studies at the lowest level to behavioral/cognitive studies at the highest level.

## 4 Genetic studies of dyslexia

A central claim of both the TSF and the NNH is that dyslexia has a neurobiological basis. Behavioral genetics—in particular twin and family studies—has long supported the conclusion that dyslexia and reading ability are heritable traits, with a significant proportion of the population variance explainable by genetic contributions (Schumacher et al., 2007). However, molecular genetics studies have not isolated one or two “dyslexia genes” that fully explain the genetic component of reading ability, but rather a variety of genes and/or chromosome regions that each have a small effect on outcomes. Consolidating knowledge on the major genes which have been implicated suggests that dyslexia may be associated with genetic disruptions to neuronal migration and cell-cell communications; in particular axonal/dendritic growth and cilia development (for reviews, see Schumacher et al., 2007; and more recently Kere, 2014).

One of these major genes is DCDC2, which is known for its effect on neuronal migration in animal models. In humans, DCDC2 has been associated with dyslexia risk and abnormal white matter integrity [reduced fractional anisotropy (FA)] in both the arcuate fasciculus (connecting Broca and Wernicke's areas) and the corpus callosum (Marino et al., 2014). At the cortical level, genetic markers from 2 major loci (DYX2, DYX3) previously associated with dyslexia have been shown to also be associated with cortical thickness and volume (Eicher et al., 2016). These included a marker in KIAA0319, which was found to be associated with cortical thickness in the left orbitofrontal region; and 2 markers in unidentified genes from DYX3, associated with cortical thickness in the left middle temporal gyrus and volume in the right fusiform area. The KIAA0319 marker was also found to be associated with FA in the corpus callosum, similar to DCDC2.

Over the past several years, several of these candidate genes for dyslexia have become mainstays in the literature (Mascheretti et al., 2017; Gialluisi et al., 2021), among them the ones previously mentioned (DCDC2, KIAA0319) and others such as DYX1C1 (Taipale et al., 2003) and ROBO1 (Hannula-Jouppi et al., 2005; Tran et al., 2014). Coinciding with the explosion of neuroimaging and genetics methods, some researchers have begun to focus their efforts on bridging the gap between the identified genetic traits and their possible downstream effects on neural endophenotypes, behavior, and cognition (Hancock et al., 2017; Mascheretti et al., 2020). The authors of the NNH explicitly frame their proposed endophenotype of neural noise and cortical asynchrony for dyslexia as being the result of a complex interplay between genetics and environment.

The year after Hancock et al.'s publication on the NNH, a separate team came out with a theoretical proposal on mapping the relationship between genetic profiles and neural oscillations related to language processing, what they term the “language oscillogenome” (Murphy and Benítez-Burraco, 2018). The authors identify many possible genes which could have causal effects on oscillatory dynamics. These developments suggest there is continuing interest both in establishing the genetic basis of dyslexia and in characterizing biomarkers related to neural oscillations—and that future research will take on the challenge of bridging these two traditionally disparate areas together. We will now focus our attention on recent developments in neural noise and oscillation research from the perspective of the NNH, which also addresses contributions of neurotransmitter signaling and other neurochemical effects on the brain to neural synchrony.

## 5 Neurochemistry and temporal sampling: a brief review of evidence for the role of neural noise

As the NNH is a recent theoretical framework, there are fewer empirical papers structured specifically around its hypotheses in comparison to the TSF. To date, there have been no systematic reviews of experimental evidence for the NNH. Nonetheless, there are several important areas of work that address evidence for the developmental pathway outlined by Hancock et al. (2017). We will first provide a brief overview of the papers which specifically target the hypotheses outlined in the original 2017 article, and then move into a broader discussion of the possible role of neurochemistry in establishing atypical pathways for structure and function that could account for some of the deficits observed in dyslexia.

### 5.1 Current empirical evidence for the NNH

One year after the publication of the NNH paper, the same group published a follow-up empirical study on primary school children using magnetic resonance spectroscopy, or MRS. MRS is an MR-based imaging technique in which measures of metabolite concentrations in the brain can be taken *in vivo* (Duncan et al., 2014). The researchers examined the relationship of Glu concentrations to cross-modal integration during an audio-visual matching task using letters, words, and pseudowords. They found that both lower GABA and higher *N*-acetyl-aspartate (NAA) in the midline occipital cortex predicted overall faster reaction time on this task (Del Tufo et al., 2018). They reported that during integration of difficult words, children with lower choline (Cho) concentrations responded faster. Finally, and perhaps most importantly, they showed that increased Glu was associated with both poorer reading ability and slower integration for words; and that cross-modal integration speed mediated the relationship between Glu and reading ability. The authors conclude that increased glutamatergic signaling exerts effects on multisensory integration, with cortical hyperexcitability serving as a plausible mechanistic explanation for this relationship; and that multisensory integration deficits then impact reading. Cecil et al. (2021) examine whether the NNH's proposed effects of neurochemistry on neural network noise extend to executive function (EF) regions, in this case anterior cingulate cortex. They report that following an EF-based intervention, children with dyslexia showed decreased Glu, combined Glu and glutamine (Glx), and NAA concentrations, while this pattern was reversed for the typical readers. However, there were no interactions with Cho.

Older MRS studies, on which the NNH based some of its hypotheses, have previously shown that excessive neurotransmitter concentrations in areas such as the left angular gyrus and midline occipital regions (implicating Glu and Cho) are associated with impaired reading ability (reading composite scores, PA, and vocabulary) in children (Bruno et al., 2013; Pugh et al., 2014). This is true both at the group level (showing differences in NT concentrations based on reading disability status—in particular higher concentrations of Glu/Cho for children with reading disorder) and at the level of individual differences (showing a continuous relationship between metabolite concentrations and performance). Bruno et al. (2013) reported a negative correlation between phonological decoding scores and Cho concentrations in the left angular gyrus (including portions of the supramarginal and posterior superior temporal gyri) in young adults, after controlling for both age and general cognitive ability. Pugh et al. (2014) applied this method to a longitudinal pediatric sample and reported that concentrations of both Cho and Glu taken from the midline occipital lobes (including portions of the lingual gyrus, calcarine sulcus, and cuneus) were negatively correlated with reading composite scores; Glu (but not Cho) was also significantly negatively correlated with PA and vocabulary. Moreover, the correlation between Glu and reading composite scores remained at follow-up 24 months later.

Kossowski et al. (2019) came to a more critical conclusion in their own study of neurometabolite concentrations in children and adults with dyslexia. They predicted that a higher relative Glu concentration in their sample of adults and children with dyslexia would be consistent with the model described by Hancock et al. (they also specified that this would be consistent with the TSF, on the basis that the involved neurotransmitters could affect low-frequency neural entrainment). Results showed that, contrary to the predictions of the NNH, there were no main effects of dyslexia status on Glu, Cho, or GABA concentrations in either the visual or temporoparietal areas of cortex; there was a main effect of dyslexia only on total NAA in the visual cortex (readers with dyslexia < typical readers). However, there was an interaction between age and dyslexia for Cho in both visual and left temporoparietal cortex: dyslexic children had a lower absolute Cho concentration compared to control children, but this pattern did not hold among adults, who showed no effect of dyslexia status. Another paper from Tan et al. (2022) attempted to quantify neural noise via a stimulus repetition paradigm, with the expectation that excessive noise would be reflected in more variable neural encoding of, and behavioral responses to, repeated stimuli. The researchers did not find any evidence to suggest that adults with dyslexia have noisier neural representations for spoken stimuli at the behavioral or neural level. However, their sample consisted of adult university students with dyslexia, so the extent to which these results generalize to young children and pre-readers, especially given the interaction reported in Kossowski et al. (2019), is not clear.

### 5.2 Glu-GABA imbalance

Hyperexcitability and altered neuronal response dynamics during crucial developmental milestones are two possible consequences of imbalanced neurotransmitters in the immature brain. The balance between GABAergic and glutamatergic activity has been shown to play a role in the developmental properties and dynamics of gamma oscillations, as gamma activity may emerge from the balance between excitatory and inhibitory neurotransmitters (often called the Glu-GABA or E-I balance; see Singh, 2012 and Buzsáki and Wang, 2012). Developmental GABAergic dynamics associated with gamma oscillations are affected by synaptic pruning, which may decrease excitation in certain brain regions as a function of age (Cho et al., 2015). The NNH argues that a predisposition to imbalanced Glu-GABA signaling may produce excessive excitability and disrupt development of crucial E-I circuits. This E-I imbalance disrupts entrainment to auditory and other sensory information at various timescales. This “noise” could be the driving neurobiological mechanism behind the TSF. However, the extent of the findings related to neurotransmitter concentrations have yet to be fully addressed in the context of individual differences and, as with many neural markers for complex neurodevelopmental disorders, are likely not universal within the dyslexic population. In summary, abnormal neurochemical activity has the potential to exert much more extensive effects on the developing brain via activity/experience-dependent mechanisms for plasticity, as we will now briefly discuss.

### 5.3 Effects of noise in relation to neuroanatomy, myelination, and conduction velocity

Properties of various white and gray matter structures differ in those with dyslexia, especially within perisylvian language regions concentrated in temporo-parieto-occipital networks and the inferior frontal gyrus (Pugh et al., 2001; Rimrodt et al., 2010; Richlan, 2020). These differences may extend to subcortical areas such as the thalamus and striatum, which themselves are densely interconnected to cortex, implying that there may be subcortical contributions to atypical speech and language development (Krishnan et al., 2016). Reduced thalamic gray matter reported in some individuals with dyslexia may reflect abnormal subcortical “gating” of language input to perisylvian areas of cortex, although corresponding findings in the striatum have been less consistent (Jednoróg et al., 2015; Krishnan et al., 2016). In terms of connectivity and white matter structure, myelination is often implicated in establishing efficiency of communication between brain regions. Results from a paper which used a novel technique (myelin water fraction imaging) indicated that poor readers have reduced myelination in both the bilateral thalamus and internal capsule (Beaulieu et al., 2020). Atypical subcortical and cortical myelin structure may result in reduced conduction velocity, which exerts downstream effects on coupled oscillations between key brain regions in the reading/language network. From a theoretical and computational perspective, reduced myelination can be conceptualized as a “conduction delay” that affects oscillation frequency in, and functional coupling between, brain regions (Pajevic et al., 2014).

The complex relationship between neuroanatomical structure and function has not yet been fully characterized, but several overarching principles have been established. The most important to our discussion are experience-dependent effects on cortical network maturation and the impact of structure on function. The human brain undergoes vast structural changes during typical development. Some changes are due to specific, consistent exposure to environmental stimuli and are not genetically “preprogrammed”, such as reading. It is reasonable to deduce that variation in these exposures may manifest in part as differences in functional activity (Zatorre et al., 2012). For instance, one group found that correlations between functional connectivity and two measures of white matter integrity—relative anisotropy and axial diffusivity—differed as a function of dyslexia diagnosis (Richards et al., 2015). It is therefore possible that (1) impoverished processing of one or multiple hierarchical timescales in speech could constitute an experience-dependent source of plasticity, affecting structural change in the brain either in conjunction with, or because of individual genetic and environmental differences; and (2) differences in structure give rise to corresponding differences in functional activity patterns. Whether white matter structural differences constitute a mechanism for increased neural noise; are themselves caused by excess noise due to genetic and/or neurochemical differences; and whether they exert long-term effects on the dynamics of functional activity in the context of dyslexia are crucial questions for future research. This is especially true given cross-linguistic reports of altered white matter microstructure and reduced integrity in dyslexia (Niogi and McCandliss, 2006; Carter et al., 2009; Vandermosten et al., 2012; Su et al., 2018).

### 5.4 Summary

Altogether, these findings implicate a potential etiological pathway for dyslexia. The NNH provides a plausible account of how dyslexia manifests as a result of individual contributions from genes, neurochemistry (specifically a Glu-GABA excitatory-inhibitory imbalance resulting in overexcitability and neural noise), and impaired neural processing of speech. There is preliminary evidence from multiple research groups in support of a relationship between altered metabolite concentrations and outcomes in young readers. The precise nature of those differences and their effects on brain development remain to be fully characterized.

## 6 Entrainment across different frequency bands

Parsing speech requires the integration of sensory information at multiple levels (e.g., syllabic parsing must be reconciled with the lower-level detection of unique phonemes to create a coherent holistic speech percept), which can be abstracted as distinct “timescales” for processing specific acoustic features of speech (Teng et al., 2017). Slow delta and theta oscillations are thought to correspond to prosodic and syllabic rhythms, while gamma oscillations are thought to be related to phonemic parsing (Goswami, 2011; Luo and Poeppel, 2012). Events in the speech signal may initiate “phase realignment” in neural networks at specific frequency bands, enhancing speech tracking for certain acoustic features; this ability has been shown to be weakened in left auditory regions in dyslexia (Lizarazu et al., 2021a).

However, one prominent limitation of the current literature is that it is quite heterogeneous. Millman et al. (2011) note, for instance, that different research groups often adhere to differing definitions of the same frequency band. They may also utilize different methods of data collection and preprocessing. While there is some overlap and generally agreed-upon ranges for these various frequencies, the precise ranges and boundaries between different bands, and by extension the specificity of reported results to a particular range, can be fuzzy (Obleser et al., 2012). Results across papers for the same identified frequency band are therefore limited in their generalizability and replicability. This is especially true for the gamma band, which is sometimes further subdivided into low- and high-gamma, with differing functional properties ascribed to each (Millman et al., 2011; Uhlhaas et al., 2011). Low-gamma oscillations < 50 Hz (the low end of the range is most often capped at or above 20 Hz) have been proposed to be specialized for verbal processing of phonemic-rate input (Giraud and Poeppel, 2012; Kershner, 2020). Higher range gamma oscillations > 50 Hz have been shown to underlie higher-level cognitive and perceptual processes, such as feature binding and attention (Başar et al., 2000; Ray et al., 2008; Fries, 2009; Friese et al., 2012; Rudo-Hutt, 2015). In contrast, the delta and theta bands (1–4 and 4–10 Hz, respectively) are both confined to narrower ranges with greater consensus than the gamma band, although variation and overlap still exists (Goswami, 2011; Harmony, 2013; Karakaş, 2020; Gunasekaran et al., 2023). For these reasons, the specific definitions for frequency ranges used by the authors of a given paper will be made explicit when their results are discussed.

### 6.1 Syllabic and prosodic-rate (theta and delta) oscillations

Syllable onset times are a particularly salient property of speech to which humans naturally attend. When syllabic-rate temporal information is artificially removed from a speech stimulus, entrainment to the speech envelope is reduced (Doelling et al., 2014). Rhythm detection and stress perception at medium temporal rates (2–20 Hz) corresponding to stress, syllables, and sub-beat has been shown to be reduced in adults with dyslexia (Leong et al., 2011; Leong and Goswami, 2014). Dyslexic adolescents also show slightly lower 3–5 Hz amplitudes during (non-simultaneous) stimulation of the left and right ears (De Vos et al., 2017a). Furthermore, positive correlations were found between PA skills and syllabic-rate 10 Hz response amplitudes in typical, but not dyslexic, adolescents. Syllabic tracking of auditory input may therefore be an important mechanism for the development of typical PA skills.

The original TSF paper emphasized the negative impact of impaired slow-wave theta entrainment at the syllabic rate on the integration of acoustic features for phonemes (Goswami, 2011). However, there was also a proposed role for the lower-frequency delta (prosodic) rhythm. Issues with low-frequency entrainment to speech and reduced synchrony in the auditory cortex may be early signs of dyslexia: one study revealed differences in the preferred phase of entrainment in children with dyslexia compared to controls in response to rhythmic stimuli (Power et al., 2013). In Power et al. (2013), adolescents (mean age of 13–14 years) took part in a rhythmic oddball paradigm in which they listened to a repetitive 2 Hz auditory stimulus of the syllable/ba/, with or without accompanying visual stimulation. Analysis of individual differences showed that there were significant correlations between their performance on a phoneme deletion task and (1) their preferred phase of delta (~2 Hz) entrainment, as well as (2) the similarity between the stimulus and its neural response. Adolescents with higher performance on phoneme deletion tasks tended to have an earlier preferred phase in the delta band, and a stronger neural representation of the auditory stimulus. That dyslexic participants had a different preferred phase from controls in the delta band suggests that neurons attuned to prosodic cues in speech may be firing sub-optimally in these children.

Adults with dyslexia also show deficits in extracting subtle rhythmic properties of irregular acoustic signals, with controls having greater EEG coherence to irregular rhythms at 2.3–2.5 Hz compared to dyslexics (Fiveash et al., 2020). Further studies on adults with dyslexia have shown both weaker inter-trial coherence for entrainment of delta oscillations to rhythmic stressed syllables in speech during a white-noise target detection task; and reduced contingent negative variation in the delta band, thought to be a component of predictive timing (Soltész et al., 2013). These measures positively predicted participant sensitivity to syllabic stress, individual phonological abilities, and reading skills.

Another study took the novel approach of speech re-synthesis, which involves reconstructing the speech envelope from EEG data—in this case, using data from a semantically-unpredictable word listening and repetition task (Power et al., 2016). The stimulus had been *noise-vocoded*, meaning that the low-frequency speech components commonly associated with prosodic information had been selectively preserved and would need to be relied upon for encoding and reconstruction. The group with dyslexia had lower-quality reconstructions of the speech stimulus compared to both the age-matched and reading-matched group. The authors conclude that impaired prosodic encoding may have a causal relationship with the phonological deficits observed in dyslexia. Additional evidence for impaired entrainment in low-frequency delta band activity comes from Molinaro et al. (2016), who showed that dyslexic readers have impaired speech entrainment and reduced synchrony in right auditory and left inferior frontal gyri (IFG) in the 0.5–1 Hz delta range—but not the 5.8–6.3 Hz theta range—while listening to sentences, compared to age-matched controls. These effects held for both children and adults, although in children the group effect in left IFG was weaker than for adults.

### 6.2 Gamma oscillations

In addition to their proposed relevance for parsing of phonemic-rate stimuli, gamma oscillations have been linked to visual object processing, feature integration, and attention (Başar et al., 2000; Ray et al., 2008; Fries, 2009; Friese et al., 2012; Rudo-Hutt, 2015). In the context of the sensory temporal sampling hypothesis, there may be abnormal left-hemisphere specialization for gamma-band oscillations in developmental dyslexia, with some arguing that deficits in low-gamma entrainment specifically reflect impaired storage, retrieval, or processing of phonemic units (Lehongre et al., 2011; Kershner, 2020).

There is evidence for a role of 30–50 Hz gamma oscillations in language, attention, and temporal binding of sensory information. One group studying resting-state frontal gamma power in 16–36-month-old children reported positive links between individual differences in gamma power and early cognitive, attentional, and language skills (Benasich et al., 2008). In addition, children with family history for language impairment had lower frontal gamma power than the controls, suggesting a heritable link to this endophenotype. Gou et al. (2011) further demonstrated that higher temporal coordination within the gamma band at 24 and 36 months was positively correlated with performance on non-word repetition tasks at 4- and 5-year follow-ups. The resting-state designs used further suggests these differences may be detectable during early stages of development even while children are not engaged in explicit language processing.

Auditory steady-state responses (ASSRs) to amplitude modulated speech-weighted noise at the phonemic level are also atypical in adolescents with dyslexia compared to controls (De Vos et al., 2017a). The reported differences include higher 20 Hz SNRs for dyslexic adolescents; in addition, positive correlations were identified between PA and 20 Hz SNRs in dyslexic readers. A longitudinal analysis in children by the same research group showed another counterintuitive result: *increased* neural synchronization to phonemic-rate 20 Hz ASSRs (especially from the left hemisphere) between the ages of 5 and 9 was negatively associated with reading (word/pseudoword reading composite) and phonological (phoneme deletion, spoonerisms) scores at ages 7 and 9 (De Vos et al., 2017b). Children who were eventually identified as having dyslexia had greater 20 Hz responses than controls, especially at ages 7–9.

Turning to findings in older adolescents and adults, a Finnish MEG study in adults who listened to ~10 min of natural speech reported that inter-subject correlations (ISCs, which quantify the extent of shared brain activation across individuals) were reduced in the dyslexia group for the delta (0.5–4 Hz) and high gamma (55–90 Hz) bands but enhanced for the theta (4–8 Hz) and low-gamma (25–45 Hz) bands (Thiede et al., 2020). Another MEG study found that for late adolescent readers, phase-locking values in the low-gamma band range (30–45 Hz) differentiated low- and high-ability readers during a phonological congruence task (Han et al., 2012). The good readers had higher phase-locking between right auditory cortex and the right superior temporal sulcus when an incongruent terminal word was more phonologically similar to the target. This pattern was reversed for the poor readers, who instead showed greater phase-locking for the dissimilar condition. The authors interpret this as reflecting weakened phonological coding in the poor readers.

The precise nature of differences in gamma frequency bands have not been fully characterized. While some studies suggest weakened entrainment or sampling at this range is predictive of language or reading deficits (Benasich et al., 2008; Gou et al., 2011), others seem to imply the opposite (De Vos et al., 2017a,b). Such discrepancies may emerge as an artifact of the developmental age of study populations: perhaps gamma activity is more important during early preliterate stages of language development and declines in relevance thereafter. As adolescents and adults, typical readers may shift to relying more on syllabic tracking rates for processing speech-like stimuli, while readers with dyslexia persist in relying on higher, gamma-rate tracking, with detrimental effects on phonological skills and subsequent reading development (phonemic “oversampling”, Lehongre et al., 2011; Giraud and Poeppel, 2012).

### 6.3 Lateralization effects

The left and right hemispheres have been proposed to engage in preferential processing of different frequency bands (Poeppel, 2003). Functional and morphological language asymmetries biased toward left hemisphere structures are well-established in the general population (Wada et al., 1975; Pujol et al., 1999; Balsamo et al., 2002; Szaflarski et al., 2002, 2012; for a review of language network structure and function see Friederici, 2011). In the dyslexia literature, abnormalities in brain asymmetry in both cortical gray matter and white matter tracts from the language network have been documented, in both case studies/reviews (Galaburda et al., 1985; Geschwind and Galaburda, 1985; Leonard and Eckert, 2008); and cross-sectional research on dyslexia (see Duara et al., 1991; Niogi and McCandliss, 2006; Altarelli et al., 2014; Zhao et al., 2016; and Banfi et al., 2019). A recent large-scale cross-sectional study on both adults and children reported more modest effect sizes for asymmetry differences in cortical regions that are associated with phonological processing (Eckert et al., 2022). Correspondingly, one might expect there to be clear differences in cerebral asymmetry of cortical oscillations between typical and poor readers.

The same year that the original TSF paper was published, Lehongre et al. (2011) showed that ASSRs in the low-gamma 25–35 Hz range are left-lateralized in controls but bilateral in dyslexia; while at frequencies ~40 Hz in the dyslexic group, asymmetry became strongly right-lateralized; and in the controls moderate right-lateralization became apparent only in the high-gamma range (55–80 Hz). Hämäläinen et al. (2012) showed evidence for reduced phase locking to slow-wave delta modulations in white noise, localized to the right auditory cortex, when comparing dyslexic to typical readers. The dyslexic profile suggested both reduced lateralization and bilateral phase locking compared to controls. Subsequent studies have since suggested that in poor readers compared to controls, there is: greater right-hemisphere dominance of neural activity at 2 Hz (identified in the supramarginal gyrus, an area linked to processing of speech rhythm and prosody, using an fNIRS amplitude-modulated white noise paradigm; see Cutini et al., 2016); and increased synchronization to 4 Hz syllabic-rate auditory stimuli, coupled with a lack of typical right-hemisphere dominance (there were also corresponding anatomical differences, such that greater asymmetry in neural synchrony was associated with asymmetry in cortical thickness; see Lizarazu et al., 2015). Lizarazu et al. (2015) also found that controls showed a positive correlation between right-lateralization of 4 Hz synchrony and word/pseudoword reading speed. And in adults (but not children), those with stronger left-lateralization at 30 Hz were more skilled at repeating pseudowords. Moreover, phase-locking values at 30 Hz were shown to be bilateral in controls, but right-lateralized in dyslexia.

These findings are far from conclusive, as there is large heterogeneity in results across the literature and even within research groups. Millman et al. (2011) report that the results of an MEG beamformer analysis using noise-vocoded single words as stimuli are in opposition to Poeppel's “asymmetric sampling in time” hypothesis (AST; see Poeppel, 2003), with increased induced theta power at 3–6 Hz in the left temporal gyrus, increased total delta power (both evoked and induced) at 1–4 Hz in the left frontal gyrus, and increased total mid-to-high gamma (60–80 and 80–100 Hz) diffusely across the right hemisphere; there were no reported differences in “traditional” gamma (25–40 Hz) or “low” gamma (40–60 Hz). In contrast to their significant findings at 2 Hz, Cutini et al. (2016) did not find significant group effects in hemispheric laterality at the ~40 Hz rate in their amplitude-modulated white noise paradigm. Lizarazu et al. (2021b) also failed to replicate previously established group differences in lateralization of delta, theta, and gamma bands. To this point, it is possible that cortical tracking of speech in the auditory cortex may be governed by multiple mechanisms that vary across differential listening contexts (Assaneo et al., 2019). These results demonstrate that while atypical lateralization in dyslexia may be pervasive, it is not universal, and the direction of reported group effects has been inconsistent. We argue for further investigation into the correspondence between electrophysiological activation, cortical structure, and metabolic activity in the brain. There remains a pressing need to characterize the precise nature of differences in entrainment that are linked to dyslexia.

### 6.4 Cross-frequency band modulations

Evidence suggests that electrophysiological activity in lower frequency bands may modulate activity at higher frequencies (Lakatos et al., 2005, 2008; Kershner, 2020), that there are hemispheric dominance effects for hierarchical processing of different oscillatory components in speech (see the previous section, in particular Lizarazu et al., 2021b and Kershner, 2020) and that there is a predictive relationship between prosodic sensitivity, PA, and reading/spelling ability (Goswami et al., 2010). Multiple studies have found modulatory cross-band interactions during cognitive tasks. For instance, theta band phase might play a modulatory role in gamma-band synchrony between relevant brain regions during expressive language tasks, and delta phase activity may modulate theta phase activity, which in turn modulates gamma activity (Lakatos et al., 2005, 2008; Doesburg et al., 2012). However, independent contributions of phonemic entrainment to reading outcomes (not necessarily modulated by syllabic or prosodic rhythms) may still exist. In Goswami et al. (2010), phonological sensitivity showed effects on reading ability that were independent of prosodic sensitivity. Nonetheless, these frequency bands are inherently intertwined: if prosodic entrainment fails, it is likely to be more difficult to then extract precise syllabic structure from speech. This problem becomes compounded for the recognition and identification of phonemes, which have an even finer-grained acoustic structure.

### 6.5 Summary

Group differences between controls and those with dyslexia at multiple temporal timescales for speech processing have been extensively documented. This is in direct support of the proposed pathway for the development of early language and eventual reading deficits in the TSF. The neural noise hypothesis also acknowledges the relevance of speech entrainment at periodic timescales—for prosody, stress, and even phonemes—to reading outcomes. In their paper, Hancock et al. propose that at “delta (2–4 Hz) and theta (4–7 Hz) frequencies, fluctuations in neural membrane potentials become entrained to quasi-periodic features in stimuli, for example speech stress patterns—exogenous and endogenous synchronization—are thought to form a basis for integrating and encoding sensory information over multiple time scales from slow prosodic and stress contours to more rapid changes that distinguish phonemes” (p. 439–440). We will now shift from neurophysiological to behavioral and cognitive correlates of the two theories.

## 7 Behavior and cognition

### 7.1 Auditory processing correlates of temporal sampling

The frameworks of temporal sampling and neural noise both present deficits in entrainment within the auditory modality as a primary source of impairment for dyslexia. As such, much of the task-based EEG analyses described in the previous section were conducted in the auditory modality. Some brief acknowledgment of basic behavioral differences is also warranted. Auditory processing deficits are common in dyslexia: processing of amplitude rise times in speech, prosodic sensitivity, stress perception, and reading/PA skills have been found to be predictive of one another and/or differ in dyslexia compared to controls (Goswami et al., 2010, 2016; Leong et al., 2011; Van Hirtum et al., 2019). One study using task-based fMRI found that dyslexic children did not show a response contrast between rapid and slow transitions of non-linguistic stimuli, while control children did, providing additional neurometabolic evidence for an abnormality in basic rapid auditory processing (Gaab et al., 2007). A systematic review of auditory processing deficits in dyslexia confirmed that some of the most consistent differences emerged for rise time discrimination and amplitude/frequency modulation (Hämäläinen et al., 2013); and a recent meta-analysis reported reliable processing deficits in children and adults with dyslexia indexed by the mismatch negativity response, for both linguistic and non-linguistic stimuli (Gu and Bi, 2020). Within the frameworks we have so far discussed, these deficits could be behavioral indicators of interference within the processing of the speech signal.

People with dyslexia have been shown to have speech-in-noise perception deficits (Ziegler et al., 2009; Dole et al., 2012) and issues with noise exclusion (Sperling et al., 2005). Neural synchrony may therefore be reduced when there is excessive environmental noise. Indeed, better speech-in-noise perception in young pre-readers ages 3–5 has been associated with increasing resting left-biased cortical oscillations at phonemic timescales (Thompson et al., 2016). Children with poorer performance on measures of speech-in-noise perception and reading also seem to be more vulnerable to background noise when processing speech syllables, having greater delays in auditory brainstem responses for noisy compared to non-noisy signals (Anderson et al., 2010).

Finally, one must also consider that any group-level differences in rapid auditory processing between controls and those with dyslexia are not universal, and are likely not the single source of phonological deficits in dyslexia (Studdert-Kennedy and Mody, 1995). Some researchers have also failed to show any such group differences in ERP responses when participants are processing stimuli of different speech-relevant durations, during both active and passive non-linguistic paradigms (see review by Schulte-Körne and Bruder, 2010). Rather, deficits may emerge only in specific contexts associated with stimulus complexity—a proposed endophenotype that has yet to be fully characterized. Protopapas (2014) is especially critical of an implicit assumption made by many researchers that diagnostic groups reflect homogeneous phenotypes and etiologies (in the case of dyslexia, that all or even most with reading deficits are presumed to have auditory temporal processing deficits). Additionally, deficits in the auditory modality do not provide a comprehensive picture of the possible correlates of temporal sampling, as will be discussed in the next section.

### 7.2 Visual correlates of temporal sampling

A major point of contention for theories involving temporal sampling is the translation of neural processing deficits in auditory speech sampling to behavioral deficits in the reading context. This cross-modal translation is not self-evident. Indeed, complementary temporal sampling theories in dyslexia have suggested that there could be a strong visual basis for the neural entrainment and asynchrony deficits reported (Vidyasagar, 2013, 2019; Pammer, 2014; Casini et al., 2018; Archer et al., 2020; Ronconi et al., 2020). A deficit in top-down visuospatial attentional processes could disrupt the grouping of letters into words, and may be associated with impaired gamma oscillations (Vidyasagar, 2013; for a meta-analytic review of visual-spatial attention in reading development see Gavril et al., 2021). Others have proposed that there could be a lack of attentional inhibition toward non-targets during graphemic parsing in dyslexia (Facoetti et al., 2006). There is some support for selective magnocellular-dorsal pathway deficits in poor phonological decoders that could impair sub-lexical routes for reading skill acquisition (Gori et al., 2014). Others have proposed simultaneous impaired temporal sampling in auditory language areas (resulting in poor entrainment to speech) and impaired theta oscillations in visual areas (accounting for visual magnocellular processing deficits seen in dyslexia) (Archer et al., 2020). However, aside from “pure” visual deficits for which evidence is currently inconclusive, deficits in the integration of auditory and visual information have been cited as a possible manifestation of the effects of neural noise.

### 7.3 Audiovisual and sensorimotor integration

To decode, and thus learn to read, children must acquire at a young age the grapheme-phoneme correspondence rules of their native language. It is therefore of no surprise that audiovisual (AV) integration has been cited as a possible prerequisite for the maturation of reading skills. One major meta-analysis found that across 31 studies, AV integration skills were correlated with measures of reading ability, including general reading, word recognition, and reading comprehension, across grade levels (Kavale, 1980). In dyslexia research, AV integration deficits are commonly reported (Hahn et al., 2014). Behavioral findings have now begun to converge with the neuroimaging and neural oscillation literature.

Delta and theta entrainment during AV oddball tasks is positively associated with individual differences in spoken and written language ability, and children with dyslexia have different preferred phases of entrainment (Power et al., 2012, 2013). Power et al. (2012) showed that theta-band (~4 Hz) activity in adolescent children aged 13 years entrained to the auditory component of AV stimuli, which consisted of a 2 Hz rhythmic auditory presentation of the syllable/ba/and a visual “talking head” stimulus conveying articulatory information (consistent with the pronunciation of/ba/). Comparison of the auditory-only (without the “talking head” stimulus) and AV conditions revealed that in typically-developing children, the presence of visual stimuli resulted in a different phase of entrainment for the auditory component. This effect was also confirmed in Power et al. (2013) using the same paradigm. Power et al. (2013) further reported an additional modulatory effect of visual cues on overall power in both the theta (~4 Hz) and delta (~2 Hz) bands in typically developing children only: there was higher delta/theta power in the auditory-only condition compared to the AV condition. Additionally, when asked to judge whether a non-linguistic stimulus is cross-modal (tone and flash), processing speed for a visual target (a flash) increases only in typical, but not impaired, adult readers when an auditory cue (a tone) is present (Sela, 2014). Multimodal cues may be serving an important role in the processing of rhythmic speech and non-speech stimuli, facilitating the processing of target input. Taken together, these results suggest that there is a unique AV interaction effect on oscillatory dynamics during cross-modal integration of auditory and visual stimuli (Schroeder et al., 2008; Power et al., 2012, 2013).

The ability to synchronize one's movements to an external rhythm has been associated with the ability to successfully encode speech at the neural level and may even be predictive of reading achievement. Carr et al. (2014) showed that reading-readiness and speech encoding in preschoolers can be predicted by their ability to physically synchronize to a beat. “Synchronizers” scored better at tests of PA, auditory short-term memory tasks, and were quicker when naming objects and colors than non-synchronizers; their auditory brainstem responses also revealed more precise neural encoding of syllables. Another electrophysiological study on beat synchronization in children with dyslexia found that ASSRs taken during passive auditory entrainment yielded significant group differences at ~2.4 Hz (Colling et al., 2017). While group differences in isolated motor entrainment were non-significant, the researchers saw evidence of abnormal sensorimotor integration at 3.6 Hz in the children with dyslexia while they tapped along to the beat (for more detail on sensorimotor synchronization issues in dyslexia and its overlap with language disorders, see the section on Outstanding Questions). On its own, a failure to physically synchronize to a beat could be attributable solely to impaired auditory processing, or to a specific sensorimotor integration deficit—however considering previous results in AV modalities, it is plausible that these deficits could be the result of issues with more generalized multimodal integration processes.

Turning to the overarching framework of sensory temporal sampling, AV integration skills could play an important role in establishing the strong multimodal language percepts which facilitate reading. Deficits in AV integration could originate from more generalizable disruptions in multisensory integration caused by sensitivity to excess extrinsic and intrinsic noise: the construction of integrated, multimodal perceptual skills and abilities requires cortical networks for the various involved sensory systems to become tuned to one another (Hancock et al., 2017). An inability to either attend to key information in one's environment (extrinsic) or an endogenous endophenotype of hyperexcitability as proposed by the NNH (intrinsic) could explain the observed phenomenon of disrupted AV integration and sensorimotor synchronization abnormalities in dyslexia.

### 7.4 Summary

Entrainment and behavioral deficits in dyslexia go beyond single-modality auditory deficits, extending to the sensorimotor and visual domains, and possibly to multisensory integration more generally. The behavioral evidence argues that the reported deficits could be a result of a generalizable impairment in sensory integration which affects the linkage of multimodal sensory representations, such as the auditory and visual components involved in decoding; or the somatosensory and auditory components involved in tapping to a beat.

## 8 Outstanding questions

### 8.1 Do issues with sensory temporal sampling constitute a shared pathway for reading and language disorders?

Reading and language disorders (specific language impairment, or SLI) are both complex conditions associated with multifactorial cognitive deficits (Catts et al., 2005). SLI is marked by impaired language acquisition and persistent deficits in oral language skills (e.g., proper use of semantics and syntax) despite typical non-verbal cognitive abilities (Tager-Flusberg and Cooper, 1999; Catts et al., 2005).

The question of whether the causal mechanisms proposed in a sensory temporal sampling framework can account for the observed deficits and endophenotypes in both reading and language disorders is an important one with some empirical support. Auditory processing deficits are common in both language and reading disorders (Tallal and Gaab, 2006; Corriveau et al., 2007; Gaab et al., 2007; Goswami et al., 2010; Leong et al., 2011; Hämäläinen et al., 2013; Van Hirtum et al., 2019). Children with language impairments, or those who are at-risk, have been shown to have abnormalities in neural entrainment at timescales that overlap with those reported in dyslexia, like the theta and gamma bands (Heim et al., 2011; Cantiani et al., 2019). Issues with rhythmic awareness (e.g., tapping to a beat) and perception of rhythmic auditory cues has also been documented in both SLI and dyslexia (Corriveau and Goswami, 2009; Cumming et al., 2015; Goswami et al., 2016). Rapid auditory processing deficits, disrupted entrainment, and impaired rhythmic processing could explain specific and common traits to both language and literacy deficits and illuminate one major source of comorbidity between these disorders (Hämäläinen et al., 2013). Yet common substrates for language and reading disorders do not translate to identical cognitive deficits.

This issue may be addressed by adhering to a more general model of sensory temporal sampling, which allows for parallel and interacting deficits in the auditory and visual domains. If auditory sampling deficits can exist in the absence of visual processing or AV integration deficits, this may explain a specific language deficit without a comorbid diagnosis of dyslexia. At the same time, those with AV integration issues or both auditory and visual sampling deficits could have a “mixed phenotype” (comorbid RD/SLI) for both reading and language. We expect that determining how this phenotypic variation emerges from subtle low-level developmental differences will be a key area for future research.

### 8.2 What are the implications for diagnosis and/or early therapeutic interventions?

At the current time, temporal sampling and neural noise remain grounded firmly in the domain of basic research on the neurobiological substrates for dyslexia. There is a considerable degree of debate over whether neural substrates are useful in identifying the disorder; they are not currently used for doing so, and we do not suggest that this practice change any time soon. Nonetheless, gaining a better understanding of the origin of reading disorders allows us to postulate the most effective forms of intervention and to consider why some children may have worse reading outcomes or respond to interventions differently.

A common refrain in dyslexia research is that children who struggle to read have aberrant phonological representations. Effective temporal sampling of speech may be a fundamental path by which these cognitive representations are formed, and behavioral interventions which target the development of strong phonological representations—such as phonics—should therefore be the most beneficial for the largest proportion of children (Van Herck et al., 2022). Children with more severe deficits in auditory processing might not be as responsive to auditory phonics-based interventions, as suggested by Vanden Bempt et al. (2022). Others might not respond to phonics training for other reasons, such as those who rely on unique cognitive strategies for word reading, such as imageability [considered an index of orthographic-semantic (O-S) mapping; see Siegelman et al., 2020]. Future research on individual differences could, and should, examine whether those children who are less responsive to the most successful phonics-based interventions, or who seem to rely on alternative cognitive strategies, also have differing electrophysiological profiles. In this way, we may uncover distinct neural biomarkers for specific cognitive reading strategies, providing a stronger basis for early identification and targeting of children with more individualized interventions.

### 8.3 Is atypical auditory sampling universal in developmental dyslexia?

We cannot claim definitively that endophenotypes for atypical temporal sampling constitute a unique subtype of dyslexia, much less that they characterize the whole dyslexia population. This is in part because of individual differences: even within studies that show empirical support for altered temporal sampling in children or adults with reading impairment, there is often overlap in the variance between the control and experimental groups. This is true of studies in the psychological and neurosciences in general; showing that there are robust group differences in neurophysiological measures on average does not necessitate that everyone in Group A is different from everyone in Group B in the same manner and to the same degree. To this point, we emphasize that the current state of research is more supportive of there being diverse phenotypic profiles that would all be classified as “dyslexia”. Some people with dyslexia have clear deficits in temporal sampling; others do not. We therefore take the well-supported position that there are multiple etiological pathways to reading disorder, originating from different developmental trajectories but with overlapping behavioral phenotypes. This review is focused on what we consider to be a major neurodevelopmental cause for dyslexia.

### 8.4 Why don't we see broader deficits in dyslexia?

Finally, an important question for consideration is why the observed behavioral deficits we see in dyslexia are not more broadly distributed across a wide range of abilities. The hallmarks of dyslexia are specific reading deficits without broad intellectual disability, yet it would seem we have not sufficiently explained why or how the effects of neural entrainment are localized in this case to language and reading. What is the mechanistic difference between oscillations in dyslexia and in other learning or neuropsychiatric disorders where atypical oscillations are found [including ADHD, autism spectrum disorder (ASD), and schizophrenia—see Uhlhaas et al., 2008; Barry et al., 2010; McFadden et al., 2012, and Rojas and Wilson, 2014; Newson and Thiagarajan, 2019 for a review of resting-state EEG abnormalities across multiple disorders]?

This subject deserves its own thorough discussion. We will note briefly that dyslexia is more likely to affect those with other learning disorders (such as dyscalculia and dysgraphia), ADHD, ASD, and other neuropsychiatric conditions like depression and anxiety (Hendren et al., 2018). Thus, the notion that we do not see broad deficits in dyslexia is, at least in part, a fallacy which emerges from the artificial dissociation between dyslexia and its spectrum of comorbidities. It is crucial that future research focus on individual differences and the co-occurrence between dyslexia and other types of disorders to highlight possible overlap between the etiologies of neurodevelopmental and neuropsychiatric morbidity.

## 9 Conclusion

We have argued that the integrated TSF and the NNH form a more comprehensive theoretical account of the causal pathway for dyslexia than either of these two frameworks on their own, addressing both its genetic and environmental origins and some of its key neurophysiological manifestations. However, the full etiological picture of dyslexia is not yet complete (Protopapas, 2014). Some children with language disorders perform well on temporal processing tasks; some with auditory processing impairments have typical language or reading skills; and some with language or reading disorders do not have temporal processing deficits (Corriveau et al., 2007; Ramus et al., 2013). Abnormal neural oscillations are likely to remain an insufficient basis for diagnostic classification in the foreseeable future. The degree to which impaired temporal sampling can serve as a legitimate biomarker for dyslexia is called into question by reports of similar oscillatory abnormalities in other disorders. Nonetheless, the presented findings provide strength to the domain-general view of neuronal synchrony in dyslexia, as one might expect separate disorders with similar underlying neurological mechanisms of dysfunction to be more commonly comorbid, which is supported by empirical evidence. Further investigation into the precise nature of comorbidity with dyslexia is warranted. Moving forward, research on sensory temporal sampling and neural noise should address these sources of variance and their behavioral, neurobiological, and cognitive links.

## Author contributions

OL: Conceptualization, Visualization, Writing – original draft, Writing – review & editing. FH: Writing – review & editing, Conceptualization.
